# Aquatic macroinvertebrates in Madeira Island (Portugal) streams: diversity and distribution

**DOI:** 10.3897/BDJ.10.e73909

**Published:** 2022-02-18

**Authors:** Pedro M. Raposeiro, Ana Balibrea, Julie-Camile Riva, Catarina Johanna Fernandes Rodrigues Ritter, Vítor Gonçalves

**Affiliations:** 1 CIBIO, Research Center in Biodiversity and Genetic Resources, InBIO Associate Laboratory / Faculty of Sciences and Technology, University of the Azores, Ponta Delgada, Portugal CIBIO, Research Center in Biodiversity and Genetic Resources, InBIO Associate Laboratory / Faculty of Sciences and Technology, University of the Azores Ponta Delgada Portugal; 2 Département de Biologie, Chimie et Géographie, Groupe de recherche sur les environnements nordiques BORÉAS, Université du Québec à Rimouski, Québec, Canada Département de Biologie, Chimie et Géographie, Groupe de recherche sur les environnements nordiques BORÉAS, Université du Québec à Rimouski Québec Canada; 3 Faculty of Sciences and Technology, University of the Azores, Ponta Delgada, Portugal Faculty of Sciences and Technology, University of the Azores Ponta Delgada Portugal

**Keywords:** aquatic insects, oceanic islands, freshwater systems, geographical distribution

## Abstract

**Background:**

The Madeira Island (Portugal; 32°24’–33°07’N, 16°16–17°16’W; 796 km^2^) is an oceanic island located in the North Atlantic, about 980 km south of Portugal and about 700 km west of the African coast. The presence of freshwater invertebrates in oceanic islands has always raised questions concerning dispersal, colonisation and evolution. Therefore, the freshwater fauna of Madeira Island has attracted the interest of many researchers in the past, the first publications going back to the nineteenth century. Initial studies were mainly taxonomic, resulting in a checklist of the Madeira freshwater macroinvertebrates with 240 taxa. As typical from oceanic islands, freshwater invertebrates are characterised by low diversity, with some taxonomic groups absent. Although freshwater Madeiran macroinvertebrates are a well-studied group, geographical information of diversity distribution is still scarce. Therefore, more studies are needed, especially georeferenced data of diversity and distribution of macroinvertebrate assemblages, to provide valuable information for improving knowledge and the development of typologically appropriate monitoring and conservation programmes and restoration strategies for local stakeholders.

**New information:**

The results of the present study revealed 713 occurrences in 40 sampling points in Madeira Island streams. The occurrence data showed 70 different aquatic taxa belonging to 21 orders and 53 families. Amongst our occurrence data, 15 endemic taxa (22.1%) from Madeira Archipelago were found. In addition, different families of Collembola and different taxa of Copepoda (Onychiuridae, Poduridae, Isotomidae, Entomobryidae, Sminthuridae) comprised new records for the Madeira streams. Therefore, further taxonomic and ecological studies on freshwater invertebrates from Madeira Island should be done with a particular focus on these lesser-known groups. Thus, our data increase the geographical data distribution of freshwater macroinvertebrates and their diversity in Madeira Island. This database is an update of geographical information of diversity distribution of Madeira freshwater macroinvertebrates known groups. This information is essential for a better understanding of community composition, diversity, occurrence or spatial distribution, which will help explore different research questions on different research areas, such as community ecology and biogeography.

## Introduction

The native stream biodiversity in remote oceanic islands is relatively depleted, compared to mainland counterparts (Covich 2006, Raposeiro et al. 2012, Pereira et al. 2014, Gonçalves et al. 2015). Distance from continental landmasses and the open ocean act as physical barriers limiting species dispersion and colonisation of remote islands (Bilton et al. 2001, Covich 2006). Furthermore, oceanic island freshwaters ecosystems are subject to a complex interaction of multiscale insular biogeographic factors, combined with local conditions on the islands themselves (Bilton et al. 2001, MacArthur and Wilson 2001, Hughes 2005, Borges et al. 2008) that act as biogeographic filters that shape the composition and structure of their communities (Raposeiro et al. 2012). This is the case of Madeiran freshwater macroinvertebrates communities that are considered assemblages with low diversity having some taxonomic groups absent (e.g. Plecoptera and Amphipoda) and many families usually contain few genera with few or even single species (Stauder 1991, Boieiro et al. 2015).

Since the 19^th^ century, freshwater macroinvertebrates communities in Madeira Archipelago have been relatively well researched through scientific expeditions, monitoring programmes and studies on freshwater ecosystems (e.g. Malmqvist 1988, Stauder 1991, Hughes 1995, Hughes and Murray 2000, Hughes and Furse 2001, Ferreira and Weihrauch 2005). According to Hughes et al. (1998), 240 macroinvertebrate taxa were recorded for Madeira Island. Freshwater macroinvertebrates communities from Madeira Island present a high degree of endemism (25.5%) (Hughes 2006, Martín et al. 2017), particularly within the Trichoptera, Coleoptera and Hydracarina groups (Stauder 1991, Baez 1993, Hughes 2003, Vidaña 2020) when compared to continental counterparts. Moreover, over 80% of the freshwater macroinvertebrates comprises insects, of which 62.5% are Diptera (Hughes et al. 1998). Endemic taxa usually occupy many biotopes due to the absence of competitors; however, in Madeira Island, most endemisms occur in low order streams at mid-high altitude (500 m a.s.l), located in indigenous Laurel forested areas (Hughes 2006).

As in most regions of the world, freshwater ecosystems of Madeira Islands suffer from environmental degradation due to the increasing anthropogenic pressure (Hughes 2005, Borges et al. 2008, Boieiro et al. 2015). Nonetheless, these are particularly vulnerable given the inherently fragile nature of insular ecosystems, coupled with their exceptional conservation value, considering the high number of endemic species and valuable biota occurring in aquatic and associated habitats (Hughes 2005, Hughes and Malmqvist 2005, Vidaña 2020). Consequently, macroinvertebrate assemblages are used widely as a structural indicator to evaluate ecosystems ecological quality, being an essential tool to understand environment disturbance in these systems and to assess long term temporal and spatial community changes (Metcalfe 1989, Metcalfe 1994, Resh et al. 1995, Zamora-Muñoz and Alba-Tercedor 1996, Czerniawska-Kusza 2005, Hussain 2012, Zeybek et al. 2014).

Despite the extensive knowledge of Madeira freshwater macroinvertebrate communities, little is known about species distribution and its georeference in Madeira Island. Therefore, this work aims to provide insight into the freshwater macroinvertebrate’s distribution during a field campaign in Madeira Island streams with georeferenced locations since no similar datasets have been previously published for Madeira.

## Project description

### Title

Aquatic macroinvertebrates in Madeira Island (Portugal) streams: diversity and distribution

### Personnel

Pedro Raposeiro, Ana Balibrea, Julie-Camile Riva, Catarina Ritter, Vitor Gonçalves

### Study area description

**Figure 1.**
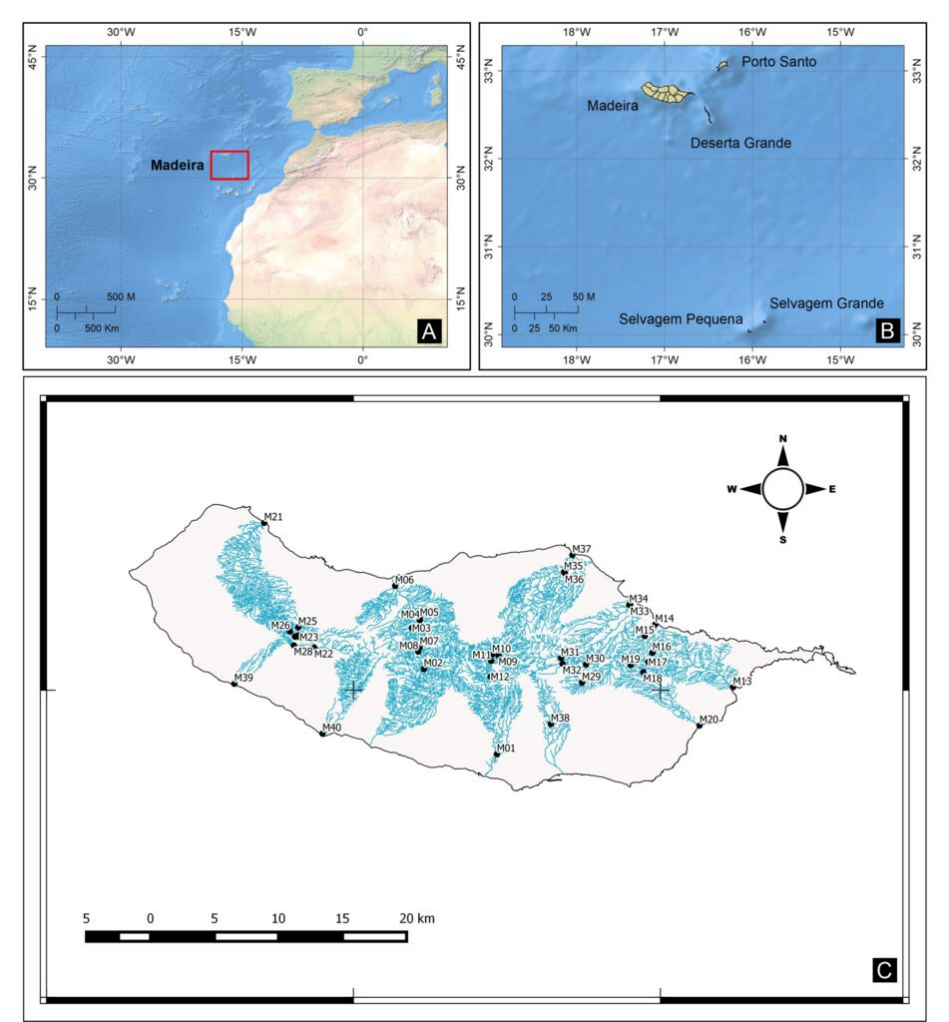
Geographical location of the study stream sites. **a** Madeira Archipelago in the Atlantic Ocean highlighted by a red square; **b** Madeira Island in the Madeira Archipelago; **c** Studied stream sites.

Madeira Island is located in the North Atlantic Ocean, 600 km west of the North Africa coast, between latitudes 32˚- 33˚ N and longitudes 16˚- 17˚ W (Fig. 1). The Island, of volcanic origin, extends for 58 km along a WNW to ESE axis and has an area of 742 km^2^ and a maximum altitude of 1861 m (Pico Ruivo).

Lying in the subtropical region, Madeira’s climate is influenced by winds from NE and the Canary Islands current. As a result, the Island has a temperate climate, characterised by mild temperatures ranging from 15.9°C in winter up to 22.3°C in summer (average annual temperature of 18.7°C) with relative humidity between 55 and 75% and annual rainfall between 500 and 1,000 mm (Santos et al. 2004).

Madeira Island presents a dense hydrographic network, comprising approximately 126 catchments and 200 streams (Marques 1994), ranging from 1^st^ to 6^th^ order. Typical for oceanic islands, streams drop strongly in altitude over very short horizontal distances, often characterised by turbulent, torrential and seasonal flow (Hughes and Malmqvist 2005, Raposeiro et al. 2013). Substrates are predominantly coarse, comprising bedrock, boulders, cobbles and sand. Due to the complex orography of the island, vegetation and land use are distributed along an altitudinal gradient. Madeira’s lower altitudes are predominantly occupied by urban and agricultural land uses, while exotic forest plantations are widespread at mid-altitudes. The native forest, Laurissilva, an essential and rare ecosystem and less impacted areas occupy most of the catchments’ higher reaches.

### Funding

This work was funded by FCT– Foundation for Science and Technology (PTDC/CTA-AMB/28511/2017 and DL57/2016/ ICETA/EEC2018/25).

## Sampling methods

### Study extent

**Figure 2.**
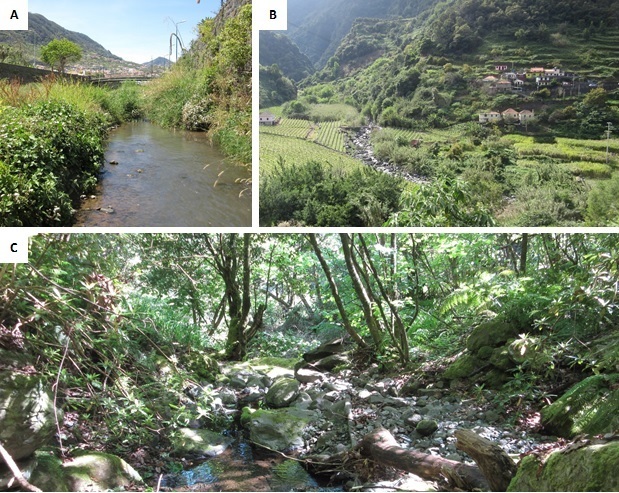
Sampling sites representing **A** lower reaches (MAD13, Ribeira do Machico); **B** middle reaches (MAD35, Ribeira de São Jorge) and **C** upper reaches (MAD30, Córrego do Arrochete).

A total of 40 sites (MAD01-MAD40) distributed by 27 permanent streams (Table 1, Fig. 1) were sampled in the spring of 2015. These sites were selected to cover a wide range of natural variation and human disturbance and ranged from low to high altitudes (Fig. 2).

### Sampling description

Benthic macroinvertebrates were collected following the national sampling protocol (INAG 2008). Benthic samples were composed of six subsamples taken with a kick-net (0.5 mm mesh) from the different existing microhabitats along a 50 m reach, preserved with 96% ethanol and transported to the laboratory. In the laboratory, samples were rinsed through a sieve of 500 μm mesh size and macroinvertebrates were sorted and preserved in 70% ethanol.

### Quality control

Macroinvertebrates were identified under a stereomicroscope (Zeiss Stemi, Deutschland). Identification was made to the lowest possible taxonomic level using identification keys (e.g. Tachet et al. 2000, Oscoz et al. 2011, Kriska 2013). Nonetheless, to facilitate understanding of results, Poduromorpha, Entomobryomorpha and Symphypleona orders were grouped in the same taxonomic group “Collembola”; Isopoda, Podocopida and Copepoda grouped as “Crustacea”; Pulmonata and Sphaeriida as “Mollusca”; Sarcoptiformes and Trombidiformes as “Acari”; Tricladida as “Platyhelminthes”; and Haplotaxida, Lumbriculida, Enchytraeida and Arhynchobdellida as “Annelida”.

### Step description

The data has been published as a Darwin Core Archive (DwC-A), which is a standardised format for sharing biodiversity data as a set of one or more data tables. The core data table contains 713 occurrences with 70 records (Raposeiro et al. 2021).

## Geographic coverage

### Description

Madeira Island, Madeira Archipelago, Macaronesia, Portugal.

### Coordinates

32.602 and 32.885 Latitude; -17.287 and -16.639 Longitude.

## Taxonomic coverage

### Taxa included

**Table:** 

Rank	Scientific Name	
kingdom	Animalia	

## Traits coverage

### Data coverage of traits

PLEASE FILL IN TRAIT INFORMATION HERE

## Temporal coverage

### Notes

2015-04-28 through 2015-05-02

## Usage licence

### Usage licence

Open Data Commons Attribution License

### IP rights notes

This work is licensed under a Creative Commons Attribution (CC-BY) 4.0 License.

## Data resources

### Data package title

Macroinvertebrates distribution in Madeira Island streams (Portugal)

### Resource link

https://www.gbif.org/dataset/bdfe1656-7b5a-4ee5-b334-72b2af17fd9d

### Alternative identifiers

http://ipt.gbif.pt/ipt/resource?r=macroinvmad

### Number of data sets

1

### Data set 1.

#### Data set name

Raposeiro P, Balibrea A, Riva J, Ritter C, Gonçalves V (2021). Macroinvertebrates distribution in Madeira Island streams (Portugal). Version 1.6. Universidade dos Açores. Occurrence dataset https://doi.org/10.15468/48axjg accessed via GBIF.org on 2021-08-25.

#### Data format

Darwin Core

#### Number of columns

33

#### Data format version

1.6

#### Description

This paper presents data from freshwater macroinvertebrate surveys developed in Madeira Island in 2015. The dataset has been published as a Darwin Core Archive (DwC-A), a standardised format for sharing biodiversity data as a set of one or more data tables. The core data table contains 40 events (eventID), 713 occurrences (occurrenceID) with 70 taxa (taxonID). The number of records in the data table is illustrated in the IPT link. This IPT archives the data and, thus, serves as the data repository. The data and resource metadata are available for download in the downloads section.

**Data set 1.** 

Column label	Column description
id	Identifier of the occurrence, unique for the dataset.
locality	Name of the locality where the event occurred.
continent	Continent of the sampling site.
country	Country of the sampling site.
islandGroup	Island group of the sampling site.
island	Island from the Island Group of the sampling site.
municipality	Name of the municipality where the event occurred.
waterBody	Water body of the sampling site.
eventID	Identifier of the event, unique for the dataset.
occurrenceID	Identifier of the record, coded as a global unique identifier.
type	The nature of the resource.
basisOfRecord	The specific nature of the data record.
eventDate	Time interval when the event occurred.
scientificName	The name with authorship applied on the first identification of the specimen.
taxonID	The identifier for the set of taxon information (data associated with the Taxon class). Specific identifier to the dataset.
Kingdom	Kingdom name.
Phylum	Phylum name.
Class	Class name.
Subclass	Subclass name.
Order	Order name.
Family	Family name.
SubFamily	Subfamily name.
Tribe	Tribe name.
Genus	Genus name.
specificEpithet	The name of the first or species epithet of the scientificName.
scientificNameAuthorship	The authorship information for the scientificName.
namePublishedInYear	The publication year of the scientificName.
taxonRank	The taxonomic rank of the most specific name in the scientificName.
decimalLatitude	The geographic latitude of the sampling site
decimalLongitude	The geographic longitude of the sampling site.
geodeticDatum	The spatial reference system upon which the geographic coordinates are based.
countryCode	Code of the country where the event occurred.
coordinateUncertaintyInMetres	The indicator for the accuracy of the coordinate location in metres, described as the radius of a circle around the stated point location.

## Additional information

### Data analysis

The multivariate analyses were performed in PRIMER v.7.0 (including the PERMANOVA plug-in) (Clarke and Gorley 2015). A resemblance matrix was formed using a Bray-Curtis distance (Clarke and Gorley 2015). Cluster analysis was used to identify macroinvertebrates assemblages and a SIMPROF test (test for the significant sign of assembly amongst samples with no pre-defined grouping) was applied to detect significant assemblages. The null hypothesis of no internal group assembly in the full set of samples was rejected when the significance level (p-value) was < 0.01. Principle Coordinates Ordination (PCO), using BrayCurtis similarity, was used to visualise the structure of macroinvertebrate assemblages.

### Results

The results of the present study revealed 713 occurrences in 40 sampling points in Madeira streams. The occurrence data showed 70 different aquatic taxa belonging to 21 orders and 53 families (Table 2).

The number and percentage composition of families and taxa under different orders are shown in Table 3. The order Diptera showed the most occurrences (36.5%) in Madeira streams, followed by Trichoptera (14.7%) and Acari (14.3%). The orders containing more families were Diptera (12 families) and Acari (8). Diptera, Coleoptera, Acari, Trichoptera and Mollusca were the more diverse aquatic macroinvertebrates orders (17, 9, 9, 8 and 8 taxa, respectively).

Chironomidae presented the highest frequency amongst aquatic macroinvertebrate families, with five taxa (Orthocladiinae, Tanypodinae, Tanytarsini, Chironomini and *Rheotanytarsus* spp.) contributing with 17.4% of the total occurrences, 7.7% from the subfamily Chironominae, 5.6% from Orthocladiinae and 4.1% from Tanypodinae. Hydroptilidae family, in the Coleoptera order, was also frequent in Madeira streams contributing with 6.7% of the occurrences and containing three taxa (*Hydroptila* spp., *Oxyethiraspinosella* McLachlan, 1884 and *Stactobia* spp.). Baetidae family, in the Ephemeroptera order, although only represented by *Baetis* spp., was also common (40 sites, contributing with 5.6%), followed by Simuliidae family (40 sites; 5.6%), belonging to Diptera order and represented by *Simulium* spp. and family Naididae (38 sites; 5.3%) from the Annelida group. Dytiscidae and Planorbidae were the families that showed higher diversity, with 4 (*Agabus* spp., Hydroporinae, *Eretessticticus* (Linnaeus,1767) and *Melademalanio* (Fabricius, 1775)) and three taxa (*Gyraulus* spp., *Planorbariuscorneuscorneus* (Linnaeus, 1758) and *Ancylusaduncus* A.A. Gould, 1847) representing each family, respectively.

Moreover, other taxa also considered most ubiquitous in Madeira streams are Orthocladiinae, Tanytarsini, *Hydroptila* spp. and Naididae presented in 40, 38, 36 and 34 sites. The mean number of taxa per sample was 18.8 ± 0.9 SE taxa. Sampling sites MAD03, MAD06, MAD16, MAD18, MAD19, MAD30, MAD34, MAD36 and MAD37, showed the highest number of taxa with 24, 23, 31, 25, 31, 27, 22, 24 and 27, respectively. In contrast, MAD01 (10 taxa), MAD05 (8 taxa), MAD06 (7 taxa) and MAD11 (10 taxa) presented the lowest number of taxa.

A total of 23 invertebrate taxa that occurred at only one to three sampling sites were considered rare. These include Diptera taxa as *Forcipomyiamadeira* Clastrier, 1991, *Rhagio* spp., Psychodidae and Anthomyiidae families. Moreover, three Coleoptera species (*Dryopsluridus* (Erichson,1847), *Eretessticticus, Melademalanio)* and three families and one subfamily of Coleoptera, Hydrophilidae, Curculionidae, Chrysomelidae and Hydroporinae were identified. In addition, a Heteroptera species *Veliamaderensis* Noualhier, 1897; two Collembola families, such as Isotomidae and Entomobrydae; the Mollusca species *Radixbalthica* (Linnaeus, 1758), *Planorbismoquini* Requien, 1848 and *Pisidium* spp.; *Arrenurusautochthonus* (Lundblad, 1942) and *Neumaniaatlantida* (Lundblad, 1941), species belonging to Acari group; and three Annelida species *Lumbriculusvariegatus* (O.F. Müller, 1774), *Fridericiabulbosa* (Rosa, 1887) and *Tubifextubifex* (O.F. Müller, 1774) were also considered as rare taxa amongst the sampled streams.

**Figure 3.**
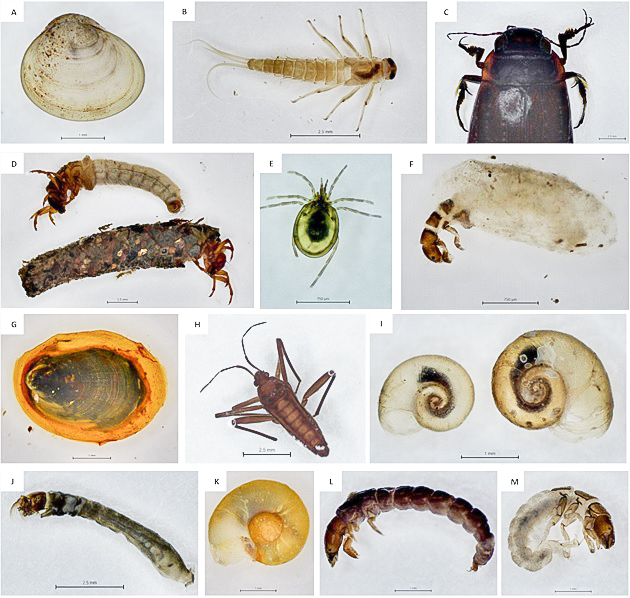
Some macroinvertebrates found from Madeira streams: **A** *Pisidium* sp.; **B** *Baetis* sp.; **C** *Melademalanio*; **D** *Limnephiluscinctus*; **E** *Torrenticola* sp.; **F** *Hydroptila* sp.; **G** *Ancylusaduncus*; **H** *Veliamaderensis*; **I** *Planorbismoquini*; **J** *Simulium* sp.; **K** *Gyraulus* sp.; **L** *Tinodes* sp.; **M** *Hydropsychemaderensis*.

Amongst our occurrence data, 15 taxa (22.1%) were described previously as endemic invertebrates of the Madeira Archipelago. The genus Baetis is represented on the Island by two endemic species, *Baetisenigmaticus* Gattolliat & Sartori, 2008 and *Baetismaderensis* (Hagen, 1865) (not distinguished in our survey) and it seems to be the most frequent endemism (present in all 40 sampling sites). *Kowarzia* and *Thaumalea* genera (Diptera) are also endemic taxa that are present in 24 and 15 studied sites, respectively. Trichoptera was the order with the higher number of endemisms, including the more common *Tinodes* spp. and *Polycentropusflavosticus* Hagen, 1865, present in 16 and 14 sites, respectively and the less frequent *Stactobia* spp. (7 sites), *Synagapetuspunctatus* (Hagen, 1859) (4 sites) and *Limnephiluscinctus* Hagen, 1865 (4 sites). Acari species, belonging to *Torrenticola, Lebertia* and *Atractides* genera, are also freshwater endemisms very common in Madeira streams, present in 22, 16 and 19 sites, respectively. Other endemic species that occasionally appeared (from 9 to 4 sampling sites) were *Ancylusaduncus* and *Agabus* spp. Moreover, the endemic Heteroptera species, *Veliamaderensis* and Coleoptera species *Melademalanio*, were considered rare endemisms because they were only present in one sampling site (MAD19 both species). Some of the taxa mentioned above, found in Madeira streams, are shown in Fig. 3.

**Figure 4.**
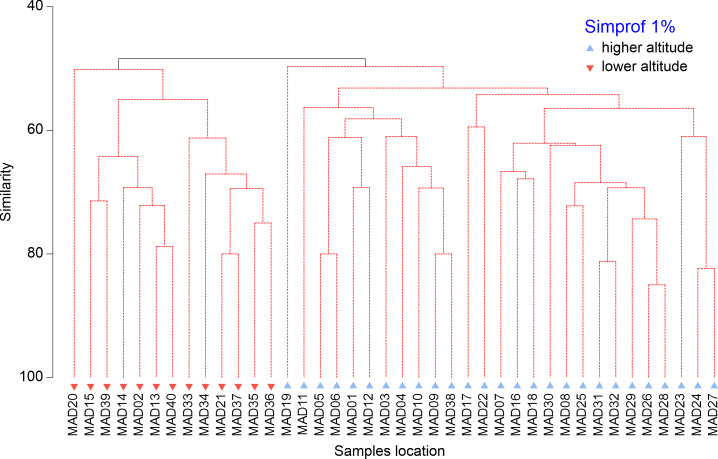
Cluster analyses of macroinvertebrate data according to their similarity. Similarity profile (SIMPROF) permutation tests were used to test for significant differences in the hierarchical cluster structure (i.e. the red dotted lines) at the 99% level.

**Figure 5.**
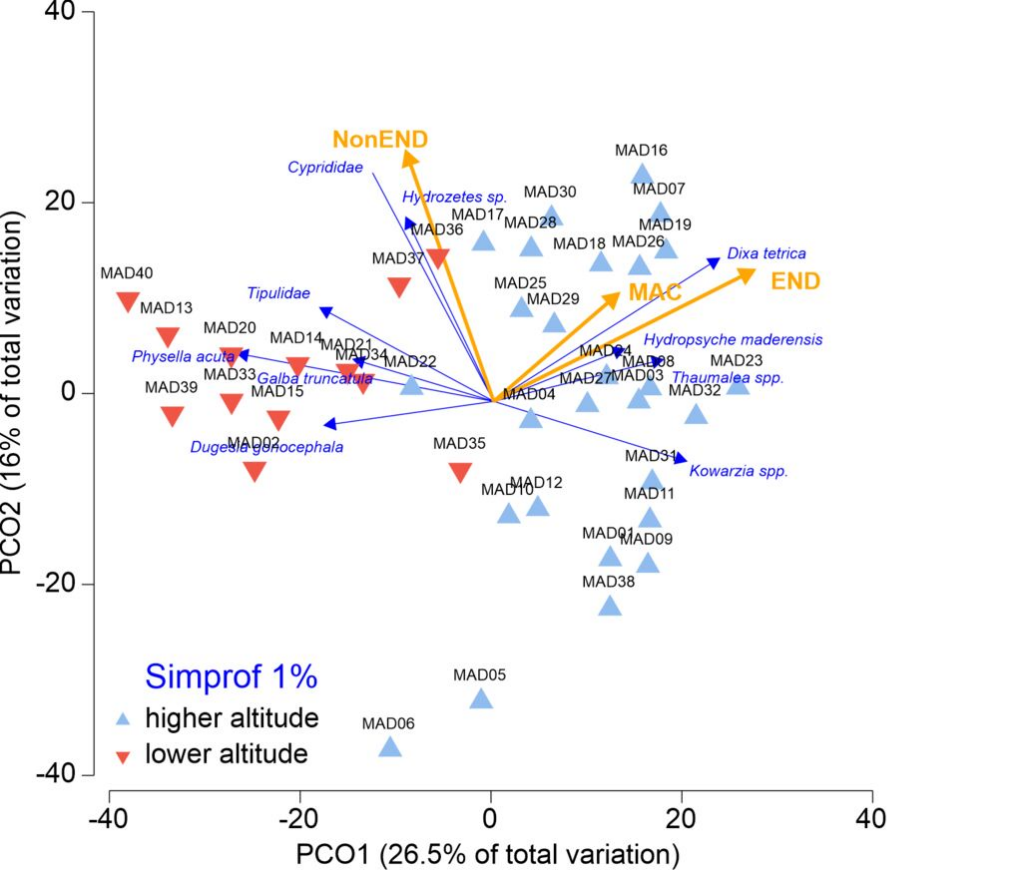
Principal Coordinate Ordination (PCO) of the first and second axes. (Non-END – non-endemic taxa; END – Endemic taxa; MAC – Endemic taxa for Macaronesia)

The cluster analysis indicated a split into two significantly different assemblages (Fig. 4, SIMPROF Global test π = 1.67, p < 0.1). SIMPER analysis revealed a dissimilarity of 51.6% between these two assemblages. The taxa that contributed most to the dissimilarity were *Physellaacuta* (4.8%), *Dugesiagonocephala* (3.9%), *Kowarzia* spp. (3.8%), *Dixatetrica* (3.5%), Cyprididae (3.3%) and *Tinodes* spp. (3.2%). The PCO analyses further supported the differences in assemblage composition between the two assemblages. The first two PCO axes explained 26.5% of total variation (Fig. 5). The first axis of the ordination (16.0% of total variation) was positively correlated to the altitudinal gradient and it separates the two different assemblages revealed by the SIMPROF. Therefore, the two macroinvertebrate assemblages are: 1) Lower altitude assemblages – most of the lower reaches located below 400 m a.s.l. These assemblages are characterised by the higher occurrence of non-endemic taxa, such as *Physellaacuta* (85%), Cyprididae (85%), *Dugesiagonocephala* (77%), *Hydrozetes* sp. (69%), *Galbatruncatula* (54%) and Tipulidae (54%) as revealed by the negative PCO1 scores. 2) Higher altitude assemblages – comprise the majority of lower reaches located above 400 m a.s.l. These assemblages are characterised by the higher occurrence of endemic taxa (endemic for Madeira and endemic for Macaronesia), such as *Kowarzia* spp. (78%), *Dixatetrica* (74%), *Thaumalea* spp. (56%) and *Hydropsychemaderensis* (56%,), as revealed by the positive PCO1 scores.

### Discussion

This study revealed how simple Madeira macroinvertebrate stream communities are compared to typologically similar continental rivers (e.g. mountain rivers), but richer when compared to other remote oceanic islands. We found 53 families of macroinvertebrates in Madeira Island streams, while Martins et al. (2020) reported 94 families of macroinvertebrates in a mainland Portuguese stream and Leunda et al. (2009) identified 74 families from a Spanish stream. Compared to even more remote oceanic islands, like the Azores Archipelago (32 families in Raposeiro et al. (2013), Ferreira et al. (2016)), Madeira Island stream communities were more diverse. This paucity of freshwater macroinvertebrates has been reported for other oceanic islands (e.g. Brasher et al. 2004, Raposeiro et al. 2012, Raposeiro et al. 2013, Balibrea et al. 2020a). However, these differences cannot draw conclusions regarding low diversity since sampling efforts used were different and have time-restricted to one season. Moreover, non-lotic systems, such as temporary or artificial ponds, were not sampled which could also contribute to the low number of aquatic macroinvertebrate taxa found (70) from the total freshwater species known to the Archipelago (240 taxa in Hughes et al. (1998)).

The most frequent macroinvertebrate taxa were from the Diptera order, especially the highly mobile taxa with multivoltine life cycle patterns, such as the Chironomidae (Berg and Hellenthal, 1992; Tokeshi, 1995). The dominance of Diptera was also reported to other oceanic islands, such as the Azores (Raposeiro et al. 2013), the Canaries (Malmqvist et al. 1993, Malmqvist et al. 1995) and Hawaii (Brasher et al. 2004). The second-largest group of stream invertebrates in Madeira Island is Acari, followed by Coleoptera and Trichoptera, which is in agreement with the total recorded freshwater aquatic fauna to the Island (Kelly et al. 2002,Borges et al. 2008).

The most well-distributed taxa on the current survey were *Baetis* spp., *Simulium* spp. and *Hydroptila* spp. The distribution of genus *Baetis* (represented by two endemic species) does not seem to be affected by local environmental factors because it was found in all 40 sampling sites. Hughes (2006) found the same pattern on some endemic trichopteran species that have extended beyond their typical ecological habitat due to the lack of competitors and trophic shift tolerance, thus spreading widely into many diverse stream habitats. This may also explain the high number of occurrences of *Simulium* spp. and *Hydroptila* spp. found in all studied sites.

Despite the large distribution of several endemic taxa, changes in the taxa occurence from the upper to lower reaches were observed jointly with a decline in endemic taxa occurrence (Fig. 5). Environmental differences between the upper and lower reaches are very distinct. Upper reaches are mainly located on the native forest with relatively undisturbed catchments (Raposeiro et al. 2020, Ritter et al. 2020). In contrast, human disturbances are much more significant in the lower reaches, where many stream banks have been changed considerably. According to several authors (França and Almeida 2003, Hughes 2005, Hughes 2006), there are significant changes from acidic, oligotrophic with low conductivity in the upper reaches to basic, meso-eutrophic with an increase in conductivity to lower reaches. The longitudinal physicochemical gradients are consistent with findings of several studies that illustrate change along the river continuum in association with altitude, land use and energy input (Vannote et al. 1980, Harding et al. 1999, Raposeiro et al. 2013, Gonçalves et al. 2015) and, thus, assemblages of macroinvertebrates are tightly associated with these water parameters (Godoy et al. 2017, Godoy et al. 2018). Significant differences in taxonomic richness amongst altitudinal and different land-use were also documented in previous studies performed on other oceanic islands (Raposeiro et al. 2013) and in continental temperate (Stone and Wallace 1998) and tropical systems (Encalada et al. 2010).

Even though Madeira aquatic fauna is considered well-studied due to studies done since the middle of the 19^th^ century on different groups of invertebrate inhabiting island freshwaters (e.g. Wollaston 1854, Hagen 1865, Eaton 1873, McLachlan 1882, Puton 1889, Reuter 1890, Hughes et al. 1998), little is known for some taxonomic groups. For example, different families of Collembola (Onychiuridae, Poduridae, Isotomidae, Entomobryidae, Sminthuridae) were recorded for the first time in Madeira freshwaters in the present study, as well as different taxa of Copepoda. Therefore, further taxonomic and ecological studies on freshwater invertebrates from Madeira Island should be done with a particular focus on these lesser-known groups.

### Final remarks

Due to the complexity and a wide range of freshwater habitats in the Madeira Island and large scale-effects from the Islands’ isolation and biogeographical filters (Smith et al. 2003, Covich 2006, Covich 2009), further studies done on freshwater communities may reveal new endemic species that may inhabit remote and inaccessible areas of the Island. Like all insular systems, Madeira freshwater systems are potentially highly vulnerable to invasive species due to the low levels of diversity (and therefore competitors) and the relative availability of ecological niches. The increasing connectivity of this Island with the mainland may also promote the transport accidentally or deliberately of new species (Gonçalves et al. 2008, Chainho et al. 2015, Lamelas-López et al. 2017, Balibrea et al. 2020b, Lenzner et al. 2020, Costa et al. 2021). Moreover, the effect of human activity related to freshwater resources, habitat degradation and water quality deterioration may dramatically change the invertebrate fauna of lotic ecosystems in this Archipelago (Hughes 2005, Leena et al. 2013, Leena et al. 2015). Thus, appropriate monitoring and conservation programmes should be undertaken on these delicate freshwater systems to understand communities’ distribution and dynamics better. Such knowledge implies the active collaboration between politicians, scientists and the local population.

## Figures and Tables

**Table 1.** Sampling codes, altitude, location and name of the stream of the 40 sampling sites on Madeira Island.

Code	Altitude (m a.s.l.)	Latitude	Longitude	River
MAD01	85	32.66319	-16.96062	Ribeira dos socorridos
MAD02	409	32.73395	-17.02101	Rib. Brava
MAD03	450	32.76807	-17.03053	Rib. da Vargem
MAD04	325	32.77415	-17.02446	Rib. de São Vicente
MAD05	311	32.77599	-17.02434	Rib. Grande
MAD06	60	32.80288	-17.04490	Rib. Grande
MAD07	903	32.75216	-17.02436	Rib. Brava
MAD08	833	32.74842	-17.02574	Rib. Brava
MAD09	826	32.74522	-16.95912	Rib. dos socorridos
MAD10	725	32.74572	-16.96462	Rib. da Gomeira
MAD11	780	32.74059	-16.96515	Corgo da Ribeira de Aneis
MAD12	597	32.72749	-16.96529	Rib. do Cidrão
MAD13	10	32.71876	-16.76422	Rib. do Machico
MAD14	36	32.77081	-16.82892	Rib. Juncal
MAD15	187	32.76142	-16.83762	Rib. Juncal
MAD16	560	32.74741	-16.83127	Rib. do Fail
MAD17	624	32.73962	-16.83465	Rib. do Machico
MAD18	791	32.73101	-16.83875	Rib. Primeira
MAD19	877	32.73715	-16.84929	Rib. do Machico
MAD20	7	32.68695	-16.79204	Rib. de Santa Cruz
MAD21	81	32.85522	-17.15374	Rib. da janela
MAD22	1391	32.75164	-17.11205	Rib. do Alecrim
MAD23	1135	32.7603	-17.12407	Rib. da janela
MAD24	1089	32.76077	-17.12833	Rib. da janela
MAD25	1041	32.76834	-17.12531	Rib. dos Cedros
MAD26	899	32.76503	-17.13236	Rib. da janela
MAD27	1003	32.76191	-17.12524	Rib. da janela
MAD28	1271	32.7535	-17.12897	Rib. do Alecrim
MAD29	1182	32.72254	-16.88974	Rib. Frio
MAD30	846	32.73768	-16.88639	Corrego do Arrochete
MAD31	637	32.74293	-16.9064	Rib. da Metade
MAD32	686	32.73838	-16.90569	Rib. das Lajes
MAD33	23	32.78725	-16.84971	Rib. de S. Roque do Faia
MAD34	42	32.78758	-16.85051	Rib. Seca
MAD35	103	32.81442	-16.90435	Rib. da São Jorge
MAD36	121	32.81342	-16.90399	Rib. da Fonte do Louro
MAD37	21	32.82849	-16.89779	Rib. de São Jorge
MAD38	517	32.67818	-16.91823	Rib. de Santa Luzia
MAD39	25	32.72153	-17.17844	Rib. da Fonte do Bugio
MAD40	22	32.68030	-17.10520	Rib. da Ponta do Sol

**Table 2.** Class, order, family and subordinate taxa collected at 40 sampling sites in Madeira Island streams in spring of 2015.

Class	Order	Family	Taxa
Insecta	Ephemeroptera	Baetidae	*Baetis* spp.
	Diptera	Simuliidae	*Simulium* spp.
		Chironomidae	Orthocladiinae
			Tanypodinae
			Tanytarsini
			*Rheotanytarsus* spp.
			Chironomini
		Thaumaleidae	*Thaumalea* spp.
		Dixidae	*Dixatetrica* Peus, 1934
		Empididae	*Kowarzia* spp.
		Tipulidae	Tipulidae
		Ceratopogonidae	Ceratopogoninae
			*Forcipomyiamadeira* Clastrier, 1991
		Limoniidae	Limoniidae
		Rhagionidae	*Rhagio* spp.
		Psychodidae	Psychodidae
		Ephydridae	Ephydridae
		Anthomyiidae	Anthomyiidae
	Trichoptera	Hydroptilidae	*Hydroptila* spp.
			*Oxyethiraspinosella* McLachlan, 1884
			*Stactobia* spp.
		Hydropsychidae	*Hydropsychemaderensis* Hagen, 1865
		Psychomyiidae	*Tinodes* spp.
		Polycentropodidae	*Polycentropusflavosticus* Hagen, 1865
		Glossosomatidae	*Synagapetuspunctatus* (Hagen, 1859)
		Limnephilidae	*Limnephiluscinctus* Hagen, 1865
	Coleoptera	Hydraenidae	*Ochthebius* spp.
		Hydrophilidae	Hydrophilidae
		Dryopidae	*Dryopsluridus* (Erichson,1847)
		Dytiscidae	*Agabus* spp.
			Hydroporinae
			*Eretessticticus* (Linnaeus,1767)
			*Melademalanio* (Fabricius, 1775)
		Curculionidae	Curculionidae
		Chrysomelidae	Chrysomelidae
	Odonata	Libellulidae	*Sympetrum* spp.
	Heteroptera	Veliidae	*Microvelia* spp.
			*Veliamaderensis* Noualhier, 1897
Collembola	Poduromorpha	Onychiuridae	Onychiuridae
		Poduridae	Poduridae
	Entomobryomorpha	Isotomidae	Isotomidae
		Entomobryidae	Entomobrydae
	Symphypleona	Sminthuridae	Sminthuridae
Malacostraca	Isopoda	Asellidae	Asellidae
Ostracoda	Podocopida	Cyprididae	Cyprididae
Copepoda	Copepoda		Copepoda
Gastropoda	Pulmonata	Physidae	*Physellaacuta* (Draparnaud, 1805)
		Lymnaeidae	*Galbatruncatula* (O.F.Müller, 1774)
			*Radixbalthica* (Linnaeus, 1758)
		Planorbidae	*Gyraulus* spp.
			*Planorbariuscorneuscorneus* (Linnaeus, 1758)
			*Planorbismoquini* Requien, 1848
			*Ancylusaduncus* A.A.Gould, 1847
Bivalvia	Sphaeriida	Sphaeriidae	*Pisidium* spp.
Arachnida	Sarcoptiformes	Hydrozetidae	*Hydrozetes* sp.
		Malaconothridae	*Trimalaconothrus* sp.
	Trombidiformes	Torrenticolidae	*Torrenticola* spp.
		Lebertiidae	*Lebertia* spp.
		Hygrobatidae	*Atractides* spp.
		Sperchontidae	*Sperchonbrevirostris* Koenike, 1895
		Arrenuridae	*Arrenurusautochthonus* (Lundblad, 1942)
		Trombidiformes	Trombidiformes
		Unionicolidae	*Neumaniaatlantida* (Lundblad, 1941)
Rhabditophora	Tricladida	Dugesiidae	*Dugesiagonocephala* Girard, 1851
Clitellata	Lumbriculida	Lumbriculidae	*Lumbriculusvariegatus* (O.F.Müller, 1774)
	Enchytraeida	Enchytraeidae	*Fridericiabulbosa* (Rosa, 1887)
	Haplotaxida	Lumbricidae	Lumbricidae
		Tubificidae	*Tubifextubifex* (O.F.Müller, 1774)
		Naididae	Naididae
	Arhynchobdellida	Erpobdellidae	*Dina lineata* (O.F.Müller, 1773)

**Table 3.** Percentage of total occurrences, number and contribution percentage of families and taxa in the different taxonomic groups.

Taxonomic groups	% total occurrences	no. of family	% of family	no. of taxa	% of taxa
Ephemeroptera	5.6	1	1.9	1	1.4
Diptera	36.5	12	22.6	17	24.3
Trichoptera	14.7	6	11.3	8	11.4
Coleoptera	2.5	6	11.3	9	12.9
Odonata	0.7	1	1.9	1	1.4
Heteroptera	0.7	1	1.9	2	2.9
Collembola	2.7	5	9.4	5	7.1
Crustacea	5.0	3	5.7	3	4.3
Mollusca	7.7	3	5.7	8	11.4
Acari	14.3	8	15.1	9	12.9
Platyhelminthes	1.8	1	1.9	1	1.4
Annelida	7.7	6	11.3	6	8.6
